# Associations between vitamin E status and bone mineral density in children and adolescents aged 8–19 years: Evidence based on NHANES 2005–2006, 2017–2018

**DOI:** 10.1371/journal.pone.0283127

**Published:** 2023-03-16

**Authors:** Aiyong Cui, Peilun Xiao, Zhiqiang Fan, Yuan Zeng, Hu Wang, Yan Zhuang

**Affiliations:** 1 Department of Orthopaedics, Honghui Hospital, Xi’an Jiao Tong University, Xi’an, China; 2 Department of Orthopaedics, The Fifth Affiliated Hospital of Sun Yat-Sen University, Zhuhai, Guangdong, China; Kindai University Faculty of Medicine, JAPAN

## Abstract

**Introduction:**

Bone mineral density (BMD) in adolescence is a crucial determinant in osteoporosis and fragility fractures in older age. Vitamin E is the most abundant lipid-soluble antioxidant present in the blood. However, the association of vitamin E status with BMD in children and adolescents remains unclear.

**Methods:**

We first measured the association of vitamin E status (serum α- and γ tocopherol) with BMD in children and adolescents with the National Health and Nutrition Examination Survey (NHANES). Multiple linear regression models were performed to evaluate their relationship after adjusting for a large range of covariates. Stratified analyses and interaction tests were used to explore their effects on different genders, ages, and races/ethnicities.

**Results:**

13,606 children and adolescents from NHANES (2005–2006, 2017–2018) were included in our analysis. Compared with the lowest α-tocopherol quartile, individuals in the highest α-tocopherol quartile are likelier to be Non-Hispanic White and have a higher value of poverty income ratio (PIR). They have a lower value of serum phosphorus and lumbar spine BMD. Every 1umol/L increase in serum α- and γ- tocopherol, the lumbar spine BMD decreased by -0.0016 and -0.0068 g/cm^2^. Compared with the lowest quartile serum α- and γ- tocopherol concentration, individuals in the highest quartile have a -0.0223 and -0.0329 g/cm^2^ lower mean BMD, respectively. Interaction effects suggest that the negative effect is more prominent among female youth, individuals aged 8–13 years, non-Hispanic whites, Mexican Americans, and non-Hispanic blacks.

**Conclusions:**

Our study indicates serum α- and γ-tocopherol are negatively correlated with lumbar BMD. Age, gender, and race may have a modifying effect on this relationship. Our study has an important clinical implication. A higher vitamin E status for children and adolescents could not improve BMD, even decrease BMD. More prospective research with stronger evidence is needed to verify our findings and their underlying mechanisms.

## Introduction

Osteoporosis is a condition characterized by decreased bone mass and impaired microarchitecture, causing bone pain and a higher risk of fragility fracture for older people [1]. Based on World Health Organization (WHO) standards, the global prevalence of osteoporosis among adults aged 50–59, 60–69, and 70–79 was 11.4%, 24.8, and 37.6% [2]. It is estimated that 22 million females and 5.5 million males have osteoporosis in European Union (EU), placing a significant health and economic burden on society [3]. The peak bone mass (PBM) in adolescence is a crucial determinant in the process of osteoporosis and fragility fractures in older age [4]. Evidence has shown that a 10% PBM increase was associated with a 50% decrease in fragility fracture risk in older age [5]. Boreham et al. reported that a 6.4% reduction in PBM during childhood and adolescence could double the risk of fragility fractures in adulthood [6]. Previous studies indicated that PBM formation could be influenced by genetics, diet and nutrition, physical activity, some diseases, and others [7–9].

As a lipid-soluble vitamin that cannot be synthesized in the body, vitamin E consists of two classes and eight isoforms, including tocotrienols (α, β, γ, δ) and four tocopherols (α, β, γ, δ). Alpha-tocopherol and gamma-tocopherol are two major forms in the body [10], which play a fundamental physiological role through their potent antioxidant properties [11, 12]. Vitamin E is associated with many diseases, including cancer, cardiovascular disease, renal disease, and dementia [13–16]. Basic and clinical research has also explored the association between a- and γ- tocopherol and bone metabolism but ended up with inconsistent results [17–19]. In a study conducted by Hermizi et al. [17], rats were given a 7 mg/kg dose of nicotine for the first two months and then 60 mg/kg vitamin E treatment in the following two months. The results showed that vitamin E reversed the bone loss caused by nicotine effects. Another study by Norazlina et al. revealed that vitamin E deficiency could reduce the spine bone mass in female rats [18]. Nevertheless, some animal studies also showed the negative effect of vitamin E on bone health. In a study by Fujita et al., they found that vitamin E could reduce bone mass by stimulating and enhancing the function of osteoclasts in the a-tocopherol transfer protein deficient mice [19]. Several epidemiological investigations on vitamin E and BMD have also reached different conclusions [20–22]. In a National Health and Nutrition Examination Survey (NHANES) study, Zhang et al. [20] found serum α-tocopherol was negatively associated with BMD in older Americans. In contrast, Yang et al.’s study [22] showed no relationship between dietary vitamin E intake and lumbar or femur BMD in older Scottish women. However, no study has investigated the relationship between vitamin E status and BMD in children and adolescents [23]. Therefore, our study evaluated the correlation between α- and γ-tocopherol and BMD in children and adolescents, aiming to provide more useful information for clinical and preventive purposes.

## Methods

The use of data was approved by the ethics review board of the National Center for Health Statistics. Written consent was obtained from each participant.

### Study population

**Fig 1:**
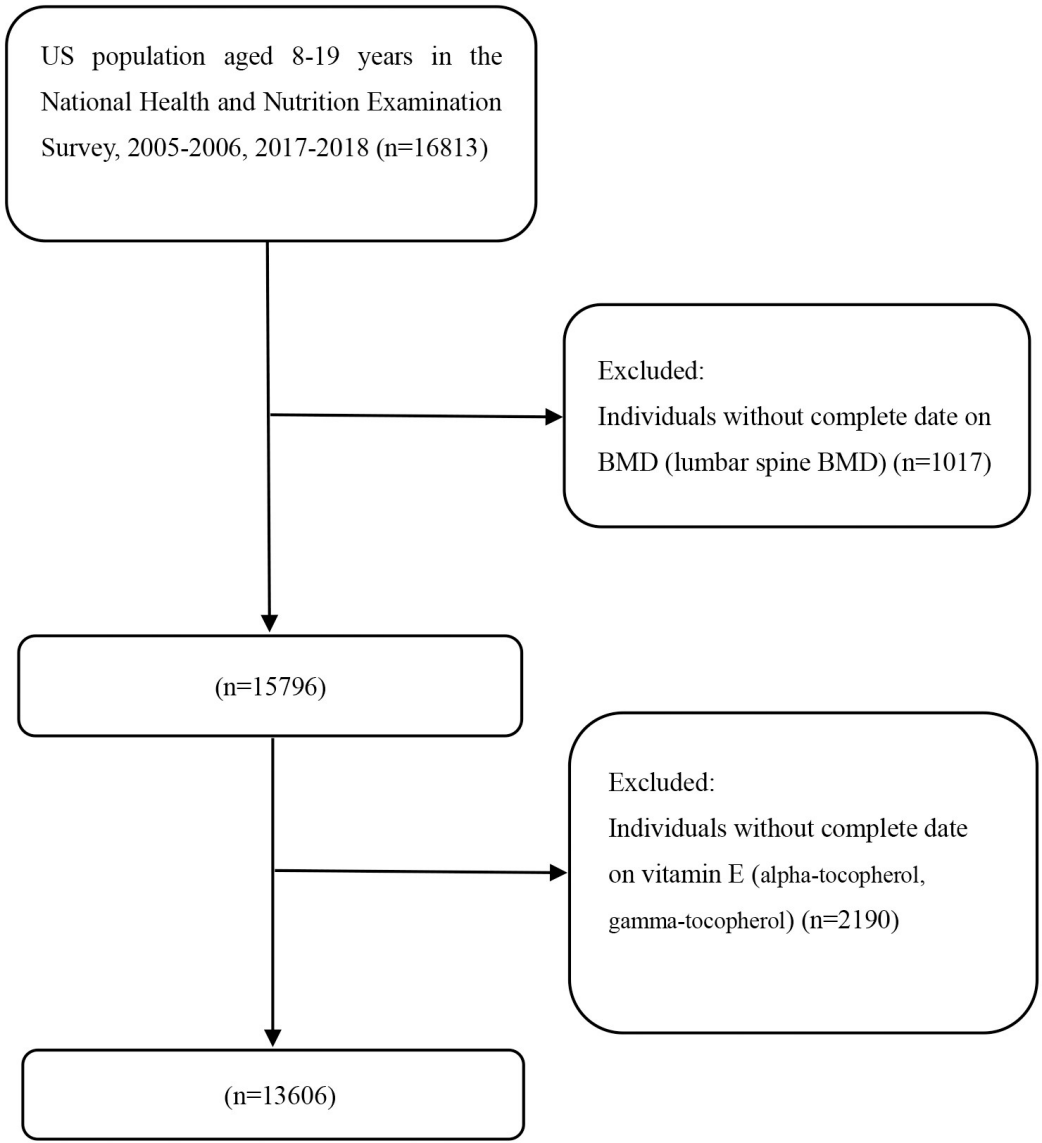
Flowchart identifying process of the NHANES participants inclusion and exclusion.

NHANES is a complex, multi-stage national nutrition survey that reflects the health status of the general U.S. population. NHANES currently collects and publicly releases data every two years. The National Center for Health Statistics (CNHS) approved all NHANES studies. Each participant signed an informed consent form in NHANES projects. We chose two cycles of NHANES (2005–2006, 2017–2018) since the data on serum vitamin E (alpha-tocopherol, gamma-tocopherol) exist only in the two cycles. Initially, 16,813 subjects aged 8–19 were found in NHANES 2005–2006 and 2017–2018. After excluding 3,207 subjects without serum vitamin E (alpha-tocopherol, gamma-tocopherol) and BMD (lumbar spine BMD), 13,606 eligible subjects are included in our analysis. Participant selection in this study is exhibited in Fig 1.

### Variables

#### Alpha- and gamma-tocopherol

The serum vitamin E concentrations (alpha- and gamma-tocopherol) were measured by modified high-performance liquid chromatography using the photodiode array detection method. The researchers mixed 100 ml of serum with an ethanol solution containing two internal standards—nonapreno-beta-carotene and retinyl butyrate. Unknown analyte quantification was completed by comparing the peak height or peak area of a known quantity of the same analyte in the calibration solution. The alpha- and gamma-tocopherol concentration was compared with retinyl butyrate at 300 nm. Serum alpha- and gamma-tocopherol information was available for individuals older than 6 in selected NHANES cycles. Detailed information on alpha- and gamma-tocopherol could be obtained from LBXVIE and LBDGTCSI datasets on the NHANES website.

#### Bone mineral density

Since the femur BMD data were only available in individuals aged over 50 years, we selected lumbar spine BMD as the dependent variable in our analyses. The data on lumbar spine BMD was available for subjects older than eight years in selected cycles, which could be obtained from the DXXLSA dataset. The lumbar spine BMD was measured by Dual-energy X-ray absorptiometry (DXA) using Hologic densitometers (Hologic, Inc., Bedford, Massachusetts) with the software Apex 3.2. The lumbar spine BMD values were collected and standardized by professionals.

#### Other covariates

Other covariates were chosen based on the published studies [8, 24, 25]. The variance inflation factor (VIF) was used to detect co-linearity between multiple variables. Covariates were excluded if the VIF >5. Concomitant covariates in this study included race/ethnicity, gender, age, PIR (poverty income ratio), body mass index (BMI), serum phosphorus, and serum calcium. A higher PIR reflects better socioeconomic status and household income. Covariates of race/ethnicity, gender, age, and PIR were collected from structured questionnaires. Other covariates on collection and processing are detailed at https://www.cdc.gov/nchs/nhanes/.

### Statistical analysis

All data and analyses were conducted with Package R 3.4 and EmpowerStats 4.0, adjusting for Mobile Examination Center (MEC) weights. The baseline characteristics of subjects were presented based on the quartile of alpha-tocopherol (Categories 1: 7.5–15.4 umol/L; Categories 2: 15.4–17.6 umol/L; Categories 3: 17.6–20.4 umol/L; Categories 4: >20.4 umol/L). We used weighted linear regression models and chi-square tests to compare continuous and categorical variables between groups. The relationship between serum α- and γ-tocopherol concentration and lumbar spine BMD was evaluated by weighted multivariate linear regression analyses. We built three models, including model 1 (unadjusted model), model 2 (adjusted for race/ethnicity, gender, and age), and model 3 (adjusted for race/ethnicity, gender, age, PIR, BMI, serum phosphorus, and serum calcium). The nonlinear association of α- and γ- tocopherol with lumbar BMD was also performed using smooth curve fits. After converting serum vitamin E concentrations to categorical variables (quartiles), another weighted multivariate linear regression model was performed. Then stratified analyses were performed by age (8–13; 14–19), gender (men, women), and race/ethnicity (Non-Hispanic White, Mexican American, Non-Hispanic Black, Other Hispanic, Other Race), and their interactions were also tested. *P* values< 0.05 were considered to be statistical significance.

## Results

Overall, 13,606 participants were enrolled in this study, with an average age of 13.59 ± 3.37. Of these participants, 59.85% are non-Hispanic white, 14.16% are non-Hispanic black, 13.09% are Mexican American, 4.99% are other Hispanic, and 7.91% are other races. The mean serum α- and γ- tocopherol concentrations are 18.95 ± 4.99 and 4.56 ± 1.94 umol/L, respectively. The weighted baseline characteristics of participants are listed based on the alpha-tocopherol quartiles (Table 1). With the exception of total serum calcium (*P* = 0.071), no statistical differences are observed in participants’ characteristics. Compared with the lowest α-tocopherol quartile, individuals in the highest α-tocopherol quartile are likelier to be Non-Hispanic White and have a higher value of PIR. They have a lower value of serum phosphorus and lumbar spine BMD.

**Table 1:** Characteristics of the study population based on alpha-tocopherol quartiles.

		Alpha-tocopherol (umol/L)					
	total		Q1 (7.5–15.4)	Q2 (15.4–17.6)	Q3 (17.6–20.4)	Q4 (>20.4)	*P* value
Number of subjects (n)	13606		3387	3396	3406	3417	
Age (years)	13.59 ± 3.37		14.11 ± 2.95	13.65 ± 3.25	13.31 ± 3.53	13.40 ± 3.55	<0.001
Gender (%)							<0.001
Men	52.11		59.92	49.09	49.39	51.23	
Women	47.89		40.08	50.91	50.61	48.77	
Race/ethnicity (%)							<0.001
Mexican American	13.09		13.26	14.36	12.87	12.16	
Other Hispanic	4.99		4.61	5.37	5.37	4.66	
Non-Hispanic White	59.85		57.65	57.23	58.02	64.94	
Non-Hispanic Black	14.16		18.65	16.28	13.74	9.73	
Other Race (Including Multi-Racial)	7.91		5.83	6.76	10.00	8.51	
BMI	22.14 ± 5.71		22.84 ± 5.84	22.12 ± 5.69	21.59 ± 5.28	22.13 ± 5.91	<0.001
PIR	2.64 ± 1.57		2.49 ± 1.56	2.58 ± 1.56	2.58 ± 1.51	2.85 ± 1.61	<0.001
Serum total calcium (mmol/L)	9.68 ± 0.25		9.66 ± 0.26	9.66 ± 0.25	9.68 ± 0.23	9.72 ± 0.25	0.071
Serum phosphorus (mmol/L)	4.44 ± 0.57		4.45 ± 0.60	4.45 ± 0.56	4.45 ± 0.56	4.42 ± 0.56	<0.001
Lumbar spine BMD (g/cm2)	0.90 ± 0.19		0.92 ± 0.18	0.91 ± 0.19	0.88 ± 0.20	0.88 ± 0.19	<0.001
Alpha-tocopherol (umol/L)	18.95 ± 4.99		-	-	-	-	-
Gamma-tocopherol (umol/L)	4.56 ± 1.94		-	-	-	-	-

Mean ± SD for continuous variables: the *P* value was calculated by the weighted linear regression model. (%) for categorical variables. The *P* value was calculated by the weighted chi-square test. Abbreviation: *BMI* body mass index. *BMD* bone mineral density. *PIR* poverty income ratio.

### Association between α-tocopherol and BMD

**Fig 2:**
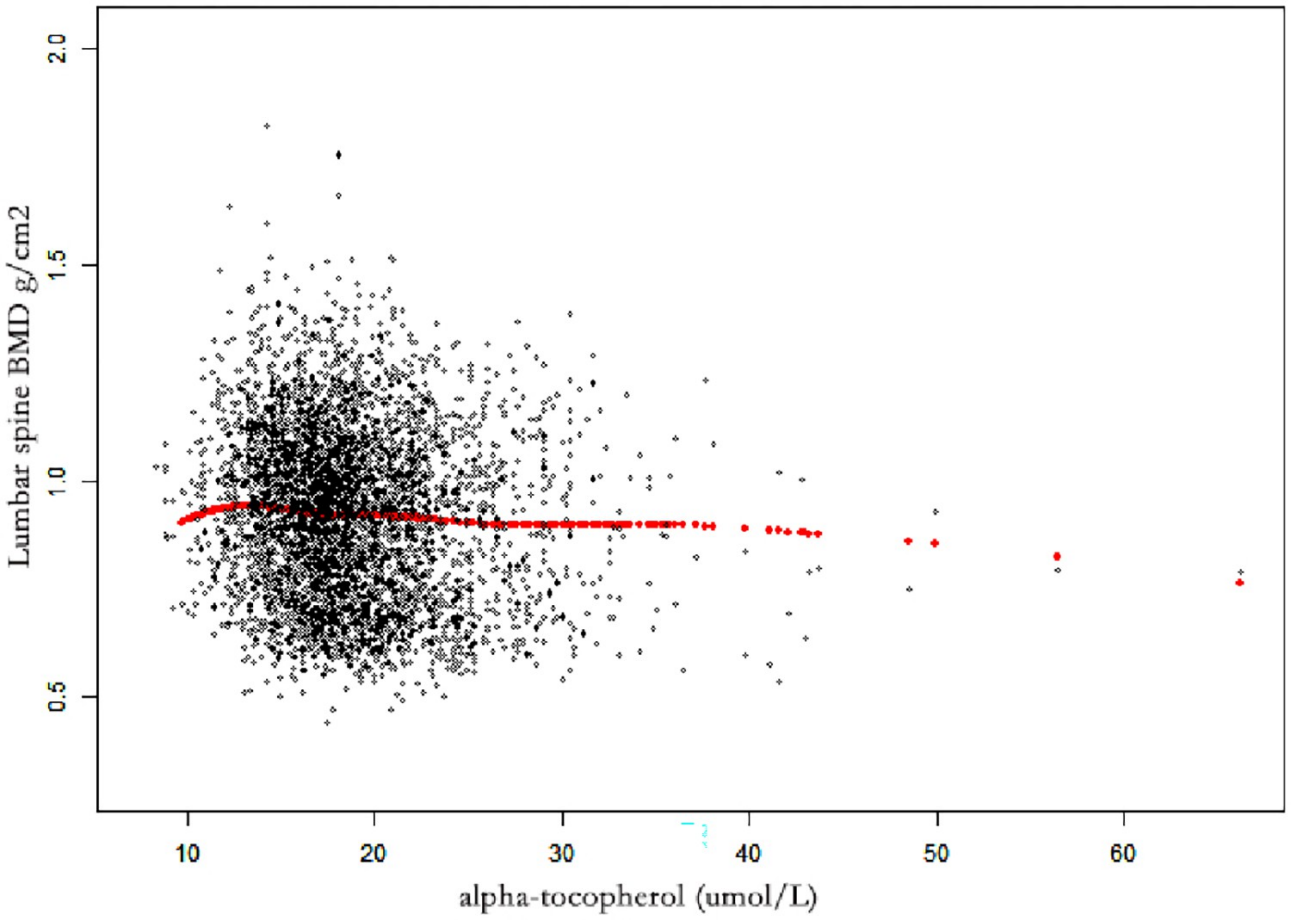
The associations between α- tocopherol and lumbar spine BMD. Each black point represents a sample. Age, gender, race/ethnicity, PIR, BMI, serum calcium, and serum phosphorus were adjusted. Abbreviations: PIR, poverty income ratio. BMI, body mass index. BMD, bone mineral density.

Table 2 shows the results of weighted multivariate regression analyses. Serum alpha-tocopherol was negatively correlated with BMD in all models. In model 3 (fully adjusted model), serum alpha-tocopherol was negatively linked with lumbar spine BMD (β = -0.0021 95% CI: -0.0025, -0.0017, *P*< 0.001) (Table 2 and Fig 2). Individuals in the highest quartile of serum α-tocopherol had a 0.0223 g/cm^2^ decreased lumbar BMD (β = -0.0223, 95% CI: -0.0279, -0.0166, *P*< 0.001) compared with the lowest quartile (Table 2).

**Table 2:** The association between serum alpha-tocopherol and lumbar spine BMD.

		Lumbar spine BMD (g/cm^2^)	
	Model 1 β (95% CI) *P* value	Model 2 β (95% CI) *P* value	Model 3 β (95% CI) *P* value
Alpha-tocopherol (umol/L)	-0.0034 (-0.0041, -0.0028) <0.001	-0.0017 (-0.0022, -0.0013) <0.001	-0.0021 (-0.0025, -0.0017) <0.001
Alpha-tocopherol categories			
Q1 (7.5–15.4)	Reference	Reference	Reference
Q2 (15.4–17.6)	-0.0126 (-0.0222, -0.0030) 0.010	0.0024 (-0.0037, 0.0085) 0.440	0.0027 (-0.0033, 0.0086) 0.380
Q3 (17.6–20.4)	-0.0434 (-0.0528, -0.0340) <0.001	-0.0123 (-0.0183, -0.0064) <0.001	-0.0119 (-0.0178, -0.0060) <0.001
Q4 (>20.4)	-0.0480 (-0.0570, -0.0389) <0.001	-0.0179 (-0.0237, -0.0122) <0.001	-0.0223 (-0.0279, -0.0166) <0.001
*P* for trend	<0.001	<0.001	<0.001
Subgroup analysis stratified by gender			
Male	-0.0037 (-0.0046, -0.0028) <0.001	-0.0003 (-0.0009, 0.0003) 0.358	-0.0008 (-0.0014, -0.0002) 0.006
Female	-0.0035 (-0.0044, -0.0027) <0.001	-0.0030 (-0.0036, -0.0025) <0.001	-0.0032 (-0.0037, -0.0026) <0.001
*P* for interaction	0.819	<0.001	<0.001
Subgroup analysis stratified by age			
8–13	-0.0038 (-0.0045, -0.0032) <0.001	-0.0032 (-0.0038, -0.0026) <0.001	-0.0027 (-0.0032, -0.0021) <0.001
14–19	0.0006 (-0.0000, 0.0012) 0.067	0.0008 (0.0001, 0.0014) 0.016	0.0002 (-0.0004, 0.0008) 0.546
*P* for interaction	<0.001	<0.001	<0.001
Subgroup analysis stratified by race/ethnicity			
Mexican American	-0.0033 (-0.0041, -0.0025) <0.001	-0.0027 (-0.0036, -0.0019) <0.001	-0.0033 (-0.0041, -0.0025) <0.001
Other Hispanic	0.0030 (0.0001, 0.0060) 0.0449	-0.0004 (-0.0021, 0.0014) 0.666	-0.0010 (-0.0027, 0.0007) 0.260
Non-Hispanic White	-0.0033 (-0.0045, -0.0022) <0.001	-0.0018 (-0.0025, -0.0010) <0.001	-0.0021 (-0.0028, -0.0013) <0.001
Non-Hispanic Black	-0.0050 (-0.0065, -0.0035) <0.001	-0.0020 (-0.0029, -0.0010) <0.001	-0.0021 (-0.0030, -0.0011) <0.001
Other races (Including multi-racial)	-0.0019 (-0.0046, 0.0007) 0.147	-0.0007 (-0.0023, 0.0009) 0.368	-0.0013 (-0.0030, 0.0003) 0.099
*P* for interaction	<0.001	0.142	0.045

Model 1: no covariates were adjusted. Model 2: age, gender, and race/ethnicity were adjusted. Model 3: age, gender, race/ethnicity, BMI, PIR, serum calcium, and serum phosphorus. Abbreviation: *BMI* body mass index. *BMD* bone mineral density. *PIR* poverty income ratio.

In stratified analysis, the negative relationship was pronounced in females (β = -0.0032, 95% CI: -0.0037, -0.0026, *P*< 0.001) than males (β = -0.0008, 95% CI: -0.0037, -0.0026, P<0.001) (*P* for interaction< 0.001) in model 3 (Table 2). The age-stratified analyses (8–13 and 14–19) showed that the negative association was more pronounced in individuals aged 8–13 (β = -0.0027, 95% CI: -0.0032, -0.0021, *P*<0.001) than those aged 14–19 (β = 0.0002, 95% CI: -0.0004, -0.0008, *P* = 0.546) (Table 2). For race/ethnicity, the negative association was pronounced in Non-Hispanic White (β = -0.0021, 95% CI: -0.0028, -0.0013, P<0.001), Non-Hispanic Black (β = -0.0021, 95% CI: -0.0030, -0.0011, P<0.001), and Mexican American (β = -0.0033, 95% CI: -0.0041, -0.0025, *P*< 0.001), but not in Other Hispanic (β = -0.0010, 95% CI: -0.0027, 0.0007, *P* = 0.260), and Other Races (β = -0.0013, 95% CI: -0.0030, 0.0003, *P* = 0.099) in the fully adjusted models (*P* for interaction = 0.045) (Table 2).

### Association between γ-tocopherol and BMD

**Fig 3:**
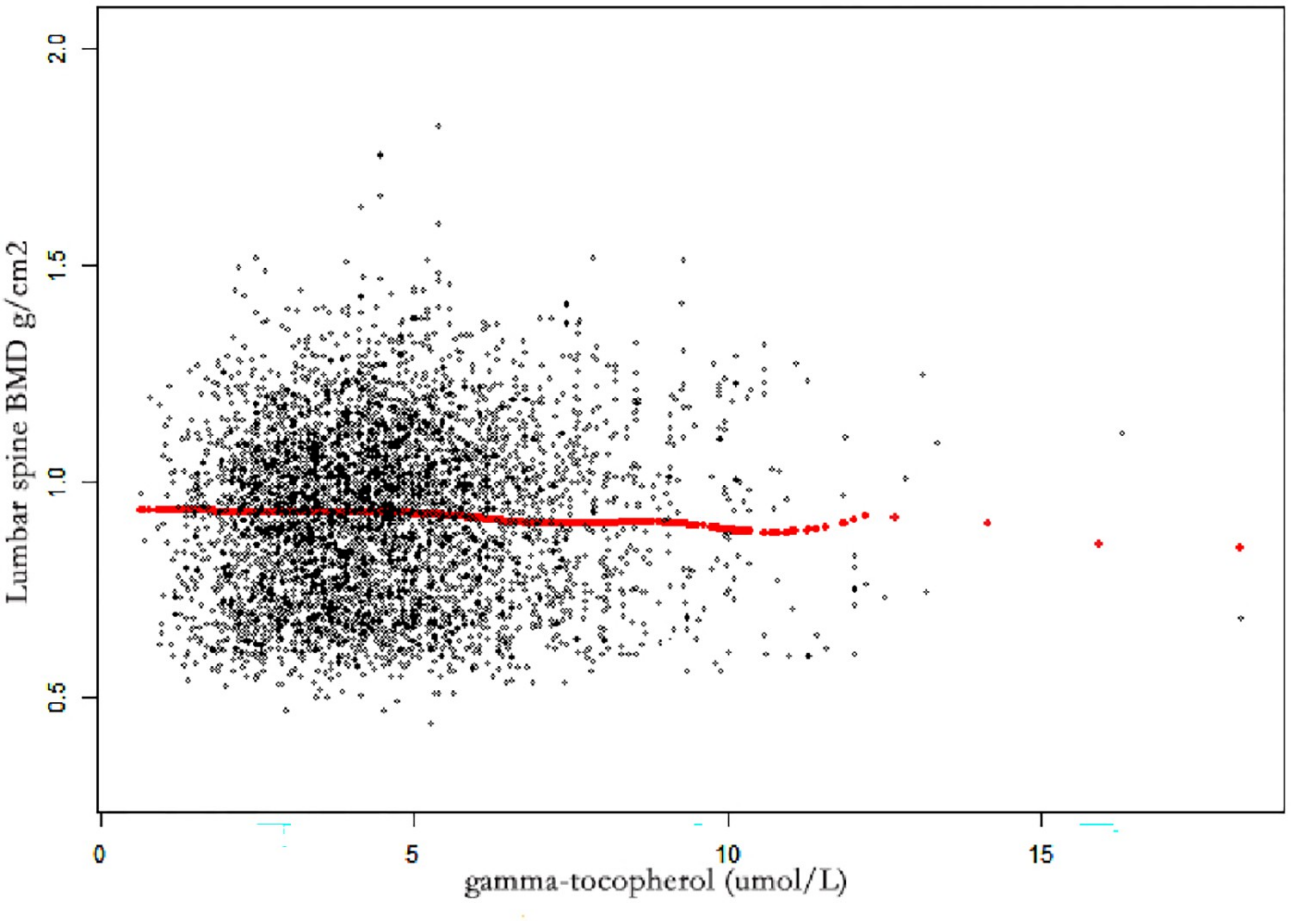
The associations between γ-tocopherol and lumbar spine BMD. Each black point represents a sample. Age, gender, race/ethnicity, PIR, BMI, serum calcium, and serum phosphorus were adjusted. Abbreviations: PIR, poverty income ratio. BMI, body mass index. BMD, bone mineral density.

In three models, serum γ-tocopherol was negatively associated with lumbar BMD (Table 3). In model 3, serum alpha-tocopherol concentration was negatively associated with lumbar spine BMD (β = -0.0068 95% CI: -0.0079, -0.0057, *P*< 0.001) (Table 3 and Fig 3). Individuals in the highest quartile of serum γ-tocopherol had a 0.0329 g/cm^2^ decreased lumbar BMD (β = -0.0329, 95% CI: -0.0387, -0.0270, *P*<0.001) compared with the lowest quartile (Table 3).

**Table 3:** The association between serum gamma-tocopherol and lumbar spine BMD.

		Lumbar spine BMD g/cm^2^	
	Model 1 β (95% CI) *P* value	Model 2 β (95% CI) *P* value	Model 3 β (95% CI) *P* value
Gamma-tocopherol (umol/L)	-0.0038 (-0.0054, -0.0021) <0.001	-0.0041 (-0.0051, -0.0030) <0.001	-0.0068 (-0.0079, -0.0057) <0.001
Gamma-tocopherol (umol/L)			
Q1 (<1.09)	reference	Reference	reference
Q2 (1.09–2.18)	0.0020 (-0.0067, 0.0107) 0.652	0.0040 (-0.0015, 0.0095) 0.153	0.0020 (-0.0034, 0.0073) 0.475
Q3 (2.18–4.25)	0.0098 (0.0009, 0.0188) 0.031	-0.0035 (-0.0092, 0.0022) 0.227	-0.0089 (-0.0145, -0.0032) 0.002
Q4 (4.25–5.00)	-0.0200 (-0.0290, -0.0109) <0.001	-0.0208 (-0.0266, -0.0151) <0.001	-0.0329 (-0.0387, -0.0270) <0.001
*P* for trend	0.001	<0.001	<0.001
Subgroup analysis stratified by gender			
Male	-0.0055 (-0.0078, -0.0031) <0.001	-0.0050 (-0.0065, -0.0035) <0.001	-0.0077 (-0.0092, -0.0062) <0.001
Female	-0.0042 (-0.0065, -0.0020) <0.001	-0.0031 (-0.0046, -0.0017) <0.001	-0.0062 (-0.0077, -0.0047) <0.001
*P* for interaction	0.461	0.034	0.061
Subgroup analysis stratified by age			
8–13	-0.0013 (-0.0029, 0.0002) 0.093	-0.0028 (-0.0043, -0.0014) <0.001	-0.0090 (-0.0105, -0.0076) <0.001
14–19	0.0013 (-0.0005, 0.0030) 0.157	-0.0011 (-0.0029, 0.0006) 0.200	-0.0036 (-0.0053, -0.0018) <0.001
*P* for interaction	0.023	0.13	0.001
Subgroup analysis stratified by race/ethnicity			
Mexican American	-0.0026 (-0.0053, 0.0001) 0.0636	-0.0001 (-0.0019, 0.0017) 0.9125	-0.0045 (-0.0063, -0.0027) <0.001
Other Hispanic	-0.0143 (-0.0235, -0.0052) 0.002	-0.0107 (-0.0162, -0.0053) <0.001	-0.0110 (-0.0170, -0.0051) <0.001
Non-Hispanic White	-0.0068 (-0.0100, -0.0037) <0.001	-0.0059 (-0.0080, -0.0039) <0.001	-0.0089 (-0.0110, -0.0068) <0.001
Non-Hispanic Black	0.0034 (0.0002, 0.0067) 0.039	-0.0019 (-0.0039, 0.0002) 0.082	-0.0032 (-0.0053, -0.0011) 0.003
Other races (Including multi-racial)	0.0032 (-0.0032, 0.0095) 0.331	0.0035 (-0.0004, 0.0073) 0.080	0.0003 (-0.0038, 0.0045) 0.870
*P* for interaction	<0.001	<0.001	<0.001

Model 1: no covariates were adjusted. Model 2: age, gender, and race/ethnicity were adjusted. Model 3: age, gender, race/ethnicity, BMI, PIR, serum calcium, and serum phosphorus. Abbreviation: *BMI* body mass index. *BMD* bone mineral density. *PIR* poverty income ratio.

The stratified analyses showed no interactive effect of γ-tocopherol on males (β = -0.0077, 95% CI: -0.0092, -0.0062, *P*< 0.001) and females (β = -0.0062, 95% CI: -0.0077, -0.0047, *P*< 0.001), (*P* for interaction = 0.061). The age-stratified analyses (8–13 and 14–19) also showed that the negative association was more pronounced in aged 8–13 (β = -0.0090, 95% CI: -0.0105, -0.0076, *P*< 0.001), but not 14–19 (β = -0.0036, 95% CI: -0.0053, -0.0018, *P*< 0.001) (*P* for interaction = 0.001) (Table 3). The race-stratified analysis showed that the negative association was more prominent in Non-Hispanic White, Mexican American, Non-Hispanic Black, and Other Hispanic, but not in Other Races in model 3 (*P* for interaction< 0.001) (Table 3).

## Discussion

Our study first evaluates the relationship between vitamin E status and BMD in children and adolescents. Our analysis indicates that serum α- and γ- tocopherol concentration is negatively related to lumbar BMD in American children and adolescents, and this association is stronger in γ-tocopherol. For every 1umol/L increase in serum α- and γ- tocopherol, the lumbar spine BMD decreased by -0.0016 and -0.0068 g/cm^2^, respectively. Individuals in the highest quartile of serum α- and γ- tocopherol concentration have a -0.0223 and -0.0329 g/cm^2^ lower mean BMD than those in the lowest quartile, respectively. In addition, the interactive effects show that the negative association between α-tocopherol and BMD is more pronounced in females, individuals aged 8–13, Mexican American, Non-Hispanic White, and Non-Hispanic Black. Furthermore, the interactive effect of γ- tocopherol with lumbar BMD also exists in different ages and races, but not sex.

In vitro studies, a- and γ- tocopherol has been proven to impact bone metabolism through their antioxidant and anti-inflammatory activities. However, the conclusions were inconsistent [19, 26–28]. Soeta et al. [28] found that α- and γ- tocopherol could decrease the activity of alkaline phosphatase of cultured osteoblasts in the rat calvariae, especially in the early stage. Nevertheless, in another study, Ahn et al. [29] found that α- tocopherol could promote osteoblastogenesis by increasing the runt-related transcription factor 2 (RUNX-2) expression. RUNX-2 has been proven to be an upstream modulator of osteoblastic genes. The potential effect of Vitamin E on bone mechanism has been explored in animals and produced diverse findings [30–32]. Feresin et al. investigated the effects of vitamin E on rats. The study indicated that medium doses (525 mg/kg diet) and high doses (750 mg/kg) of vitamin E could reverse the bone loss of ovariectomized rats [30]. Another study by Muhammad et al. showed that vitamin E treatment for ovariectomized young rats could prevent bone mass reduction by increasing trabecular volume, number, and separation [31]. In addition, an early investigation showed that vitamin E has the potential to recover bone metabolism impaired by nicotine [32]. Nevertheless, the adverse effects of vitamin E on bone metabolism were also observed in animal experiments [19, 33]. Fujita et al. [33] found that mice deficient in α-tocopherol transfer protein have a higher BMD than wild-type mice. They indicated that α-tocopherol could enhance the activity of osteoclast fusion by inducing the expression of dendritic-cell-specific transmembrane protein [33]. Wild-type mice fed a diet supplemented with a high dose of alpha-tocopherol also experienced bone loss [33]. Several aspects could explain these inconsistent results. First, the differences in age and sex of the rat may cause variations in the results. Second, the exposure doses used in these studies are also different. Previous evidence has proven that the effect of vitamin E on bone metabolism could be dose-dependent [34]. A study by Smith et al. found that treating male rats with a high dose of α-tocopherol could prevent osteoporosis induced by hindlimb-unloading, but this was not observed in a low dose of α-tocopherol. Furthermore, the large doses of tocopherol used in the rat studies far exceeded the recommended daily vitamin E consumption in humans, which makes it hard to generalize these findings in the rat model to the population [35].

Several epidemiological studies investigating the association between vitamin E and adult BMD have also drawn inconsistent results [36, 37]. In 19 years of a follow-up study in Sweden [38], 61,422 women and 1138 men were included for analysis. The results suggested that a lower α-tocopherol intake was related to a higher hip fracture rate in women. Nevertheless, serum α-tocopherol concentration was connected to decreased fracture rate in men. A recent mendelian randomization study showed that higher serum α-tocopherol was linked with a greater BMD [37]. However, Wolf et al. [36] measured 11,068 women aged 50–79, and they observed no associations between dietary intake, serum vitamin E, and BMD after adjusting an extensive range of BMD-related covariates. Similarly, in another NHANES study, Li et al. [39] found no significant associations of serum vitamin E levels with femur BMD. An early longitudinal study conducted by MacDonald et al. [40] observed a negative association between α-tocopherol intake and BMD in the femoral neck and lumbar spine in premenopausal women aged 45–54 years. However, serum α-tocopherol level was not included in this study. Vitamin E intake calculated by dietary recall only is not entirely credible. Differences in serum vitamin E levels across populations and studies may provide an explanation for these inconsistent results [22, 41, 42]. Some previous studies in adults have indicated a positive association between vitamin E and BMD in low vitamin E populations [22, 41], whereas a negative association in those with higher vitamin E levels [42]. Thus, a vitamin E excess could be the reason for the impairment of bone health.

Our study shows a stronger effect of γ- tocopherol than α- tocopherol on BMD in children and adolescents. Gamma-tocopherol is the major form of vitamin E from dietary sources, while alpha-tocopherol is the predominant form of vitamin E supplements [11, 43]. In vivo, Gamma-tocopherol showed a more potent antioxidant and anti-inflammatory ability than alpha-tocopherol [44]. Contrary to our findings, a previous study of postmenopausal women showed that higher serum α-tocopherol levels and low serum α/γ-tocopherol value were associated with elevated BAP levels (a biomarker of bone formation) [42]. Another study of an older American population showed a negative correlation between serum levels of α-tocopherol and BMD, but the negative association was not significant in γ-tocopherol. However, the youth are in a period of rapid growth and development, and the effect of vitamin E and its isomers on bone density in children may differ from that in adults. Our study was also the first to explore the association between different vitamin E isomers and bone health in children and adolescents. Further studies with stronger evidence are needed to verify our findings and their underlying mechanisms. Another notable finding in our study is that the negative effect of vitamin E is more pronounced in individuals aged 8–13 years than in 14–19 years. Previous studies showed that children and adolescents achieved the highest BMD increase rate at around 17 years in males and 15 years in females. Thus, the negative effect of vitamin E on BMD may be attenuated after reaching these ages [45]. However, due to the lack of evidence from clinical and basic studies, more prospective studies are needed to verify our speculations.

Although there are some epidemiological studies in adults, no study has investigated the relationship between vitamin E status and BMD in children and adolescents. Vitamin E is essential in children’s rapid growth and development and is more likely deficient than in adults [46]. Therefore, vitamin E’s biological effects on BMD may differ in adults and children. Our study identifies an inverse relationship between vitamin E and BMD in children and adolescents. Vitamin E supplementation during childhood may be helpful in issues like lung function, kwashiorkor, or liver disease [47–49]. Vitamin E supplementation for children and adolescents could not improve bone health or even damage bone health.

Our study has some advantages. First, this study is conducted based on NHANES, and all analyses used MEC weights, which can be representative of the general U.S. population. Second, we selected serum α- and γ-tocopherol rather than dietary vitamin E intake as exposure variables since they could better reflect an individual’s vitamin E status [50]. Some potential limitations in this study should also be acknowledged. First, no causal association could be inferred because of the nature of the observational study. Second, we could not investigate the relationship between vitamin E and femur BMD since the lack of data in NHANES. However, studies have shown that the relationship between vitamin E and BMD may be site-specific. For instance, Mata-Granados et al. [41] evaluated the relationship between vitamin E status and osteoporosis in Spanish postmenopausal women. They found that vitamin E was positively associated with lumbar spine BMD but not femur BMD. It may be attributed to the lumbar spine having more trabecular bone than the femur. Additional studies are required to explore the association between vitamin E and BMD at various sites. Third, we did not investigate the association in children younger than eight years old since the limitation of the NHANES database. More research should be carried out to explore the association between vitamin E status and BMD in children under eight years.

## Conclusions

Our study is the first study that explores the association between vitamin E and BMD in children and adolescents. The results indicate that serum α- and γ-tocopherol are negatively correlated with lumbar BMD. Age, gender, and race have a modifying effect on this relationship. Our study has important clinical implications. A higher vitamin E status for children and adolescents could not improve bone health and even damage bone health. In addition, more prospective research with more robust evidence is needed to verify our findings and their underlying mechanisms.

## List of abbreviations

BMD: bone mineral density
PIR: poverty income ratio
WHO: World Health Organization
PBM: peak bone mass
NHANES: National Health and Nutrition Examination Survey
NCHS: National Center for Health Statistics
DXA: dual-energy X-ray absorptiometry
VIF: variance inflation facto
BMI: body mass index
RUNX-2: runt-related transcription factor 2
MSC: Swedish Mammography Cohort
ULSAM: Uppsala Longitudinal Study of Adult Men
